# A polypeptide from the junction region sequence of EWS-FLI1 inhibits Ewing’s sarcoma cells, interacts with the EWS-FLI1 and partner proteins

**DOI:** 10.1038/s41598-017-07482-4

**Published:** 2017-08-03

**Authors:** Krishna Priya Thangaretnam, Gopal Gopisetty, Priya Ramanathan, Thangarajan Rajkumar

**Affiliations:** Department of Molecular Oncology, Cancer Institute (WIA), Chennai, India

## Abstract

The EWS-FLI1 chimeric protein uniquely expressed in Ewing’s sarcoma has an obligate role in its aetiology. In our previous report we showed that ectopic expression of the DNA sequences form the junction region (a.a 251–280) can inhibit Ewing’s sarcoma cell growth. In the present report, we introduced a peptide (TAT/NLS/EWS-PEP) comprising of thirty amino acids spanning the junction in conjunction with HIV-1-trans-activating (TAT) and nuclear localization signal sequence (NLS). Peptide uptake and localization studies revealed presence of peptide in ~99% of transduced cells and in the nucleus. Peptide transfection induced cytotoxicity relative to untreated and TAT-NLS peptide treated Ewing’s sarcoma cells. The peptide inhibited clonogenicity, cell cycle, bromo-deoxy uridine (BrdU) uptake and invasion capacity of treated cells. The treatment also affected epithelial to mesenchymal transition (EMT) markers and EWS-FLI1 target gene expression levels. Co-immunoprecipitation experiments involving ectopically expressed full-length EWS-FLI1 protein and the peptide revealed an interaction. Additionally, we found that peptide interaction also occurs with the protein-GGAA microsatellite sequences complex known to contain EWS-FLI1. Further, in the pull-down assay, the peptide was found to interact with proteins known to potentially interact with EWS-FLI1. Based on these results we conclude that peptide could be applied in targeting EWS-FLI1 protein.

## Introduction

Ewing’s sarcoma is a highly aggressive malignant bone and soft tissue tumour, seen in children and young adults. Ewing’s sarcoma treatment combines surgical and/or radiation therapeutic approaches for local control along with chemotherapy for systemic control of disease. Despite optimal management, and increase in the survival rate for localized disease, treatment response in metastatic disease at presentation has a poorer outcome; therefore there is a need for treatment approaches to be explored to complement/increase the effectiveness of available treatment modalities^1^. A defining feature of the malignant cells is the presence of a translocation, between the central exons of the EWSR1 gene (Ewing Sarcoma breakpoint region 1; chromosome 22) to the central exons of an ets family gene; frequently FLI1 (Friend Leukaemia Integration 1; chromosome11) or ERG (v-ets erythroblastosis virus E26 oncogene homolog; chromosome 21) t(11;22) and t(21;22), respectively. The EWS contributes to the transactivation domain, while the FLI1 contributes to the DNA binding domain and the chimeric protein functions as a transcription factor^2^. EWS-FLI1 is an intrinsically disordered chimeric protein that has been shown to induce tumorigenesis and is critical to the maintenance of the malignant phenotype^3–5^. Previously, it was shown that the activity of EWS-FLI1 protein can be inhibited using small molecule and peptides^6, 7^. The peptides were derived from the sequences of the interacting protein partners or from phage display which identified novel peptides interacting with the EWS-FLI1 protein.

In our previous report we had demonstrated that sequences derived from the junction region (a.a. 251–280) of EWS-FLI1 protein when expressed in Ewing’s sarcoma cells inhibited their tumorigenic properties, and affected epithelial to mesenchymal transition (EMT) markers and EWS-FLI1 target genes expression^8^. In the present report we show that a peptide derived from a combination of amino acid sequence from the junction region (a.a. 251–280) along with NLS and HIV-1-trans-activating (TAT) protein sequence localizes to the nucleus and inhibits the growth properties of cells. We show that the peptide can interact with the EWS-FLI1 complex, GGAA nucleotide protein complex known to contain EWS-FLI1 protein, and proteins known to potentially interact with EWS-FLI1.

## Results

### Cell Penetration and Localization of Peptides

**Figure 1:**
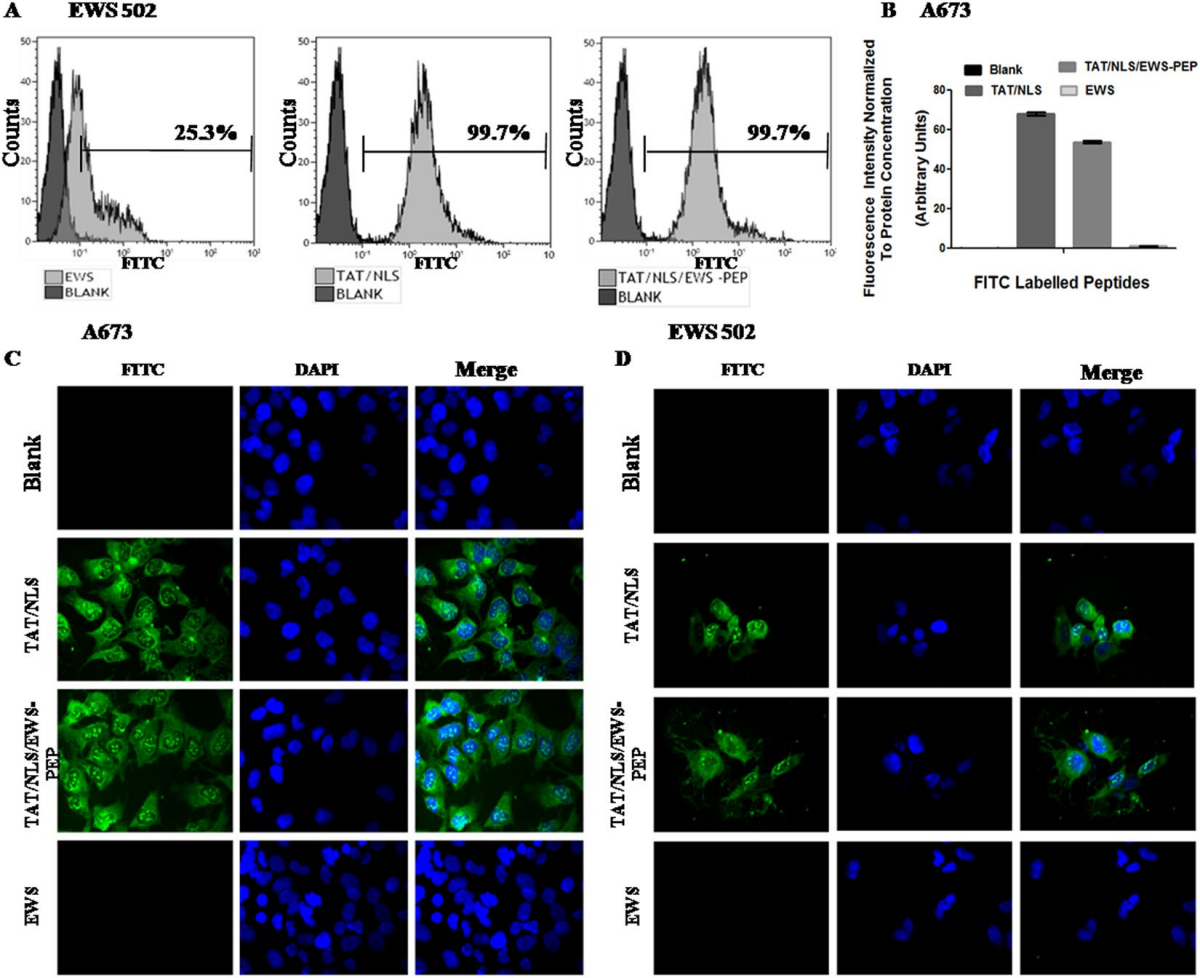
Uptake and localization of peptides in Ewing’s sarcoma cells. (**A**) FACS analysis of EWS502 cells treated with (grey) or without (black) FITC labelled peptides. Percentage above the gate represents FITC positive population. (**B**) FITC Fluorescence intensity of A673 cell lysates, either untreated or treated with FITC labelled peptides. (**C**) Fluorescence images of A673 cells either untreated or treated with FITC labelled peptides, captured at 40× magnification. (**D**) Fluorescence images of EWS 502 cells, untreated or treated with FITC labelled peptides.

For this study we used three different peptides (Supplementary Table 1). Peptide EWS-PEP comprised of 30 amino acids spanning 15 a.a. from the EWS portion and 15 a.a. from the FLI1 portion located on either side of the fusion region of the EWS-FLI1 protein. Another peptide (TAT/NLS) comprised a combination of sequences of HIV-tat cell penetrating peptide along with NLS sequence for nuclear localization. The final peptide (TAT/NLS/EWS-PEP, designated CIEWSPEP)^9^ comprised of TAT and NLS sequence at the N terminal followed by the EWS-PEP peptide sequence. Peptide uptake and localization studies using N-Terminal FITC labelled peptides showed that the uptake of the peptides TAT/NLS and TAT/NLS/EWS-PEP was 99.7% whereas EWS-PEP peptide uptake was detected only in 25.3% of EWS502 cells relative to untreated cells (Fig. 1A). The cell penetration was further confirmed by measuring the intracellular and nuclear fluorescence following cell lysis. The fluorescence normalized to total protein concentration reflected the increased uptake of both TAT/NLS (68.12 a.u.) and TAT/NLS/EWS-PEP (53.83 a.u.) relative to blank (0.10) or EWS-PEP (1.18 a.u.), (Fig. 1B). Next, the fluorescence microscopy analysis of A673 and EWS502 cells revealed cell penetration, cytoplasmic and nuclear localization in TAT/NLS and TAT/NLS/EWS-PEP treated cells relative to untreated cells (Fig. 1C and D). However, the EWS-PEP peptide did not reveal overt cell penetration or nuclear localization. Since the uptake of EWS-PEP peptide was limited subsequent experiments were performed using TAT/NLS and TAT/NLS/EWS-PEP.

**Figure 2:**
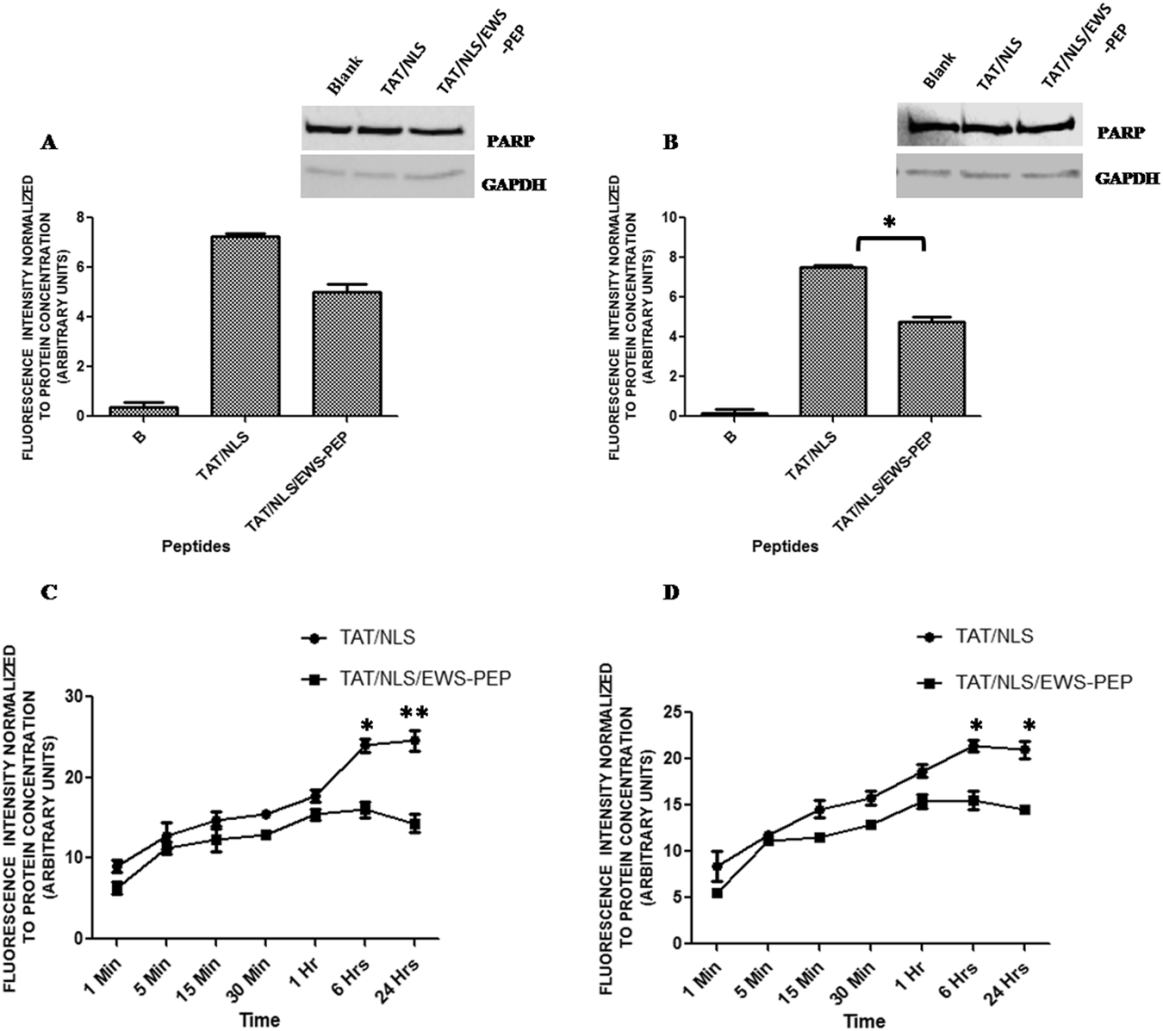
Nuclear Localization and Time-course uptake of peptides. Fluorescence intensity of the nuclear extracts from untreated and peptide treated A673 (**A**) and EWS502 cells (**B**). The western blot inserts in both the graphs depicts the quality of the nuclear extracts, with PARP being used as nuclear marker and GAPDH as cytoplasmic marker. The cropped blots are displayed, for full blots refer supplementary information. Time dependent uptake of fluorescently labelled peptides in A673 (**C**) and EWS502 (D) cells. *p < 0.05, **p < 0.01.

In the case of nuclear extracts also, increase in fluorescence of both TAT/NLS (7.22 a.u in A673 and 7.52 a.u in EWS502) and TAT/NLS/EWS-PEP (5.01 a.u in A673 and 4.77 a.u in EWS502.) relative to blank (0.37 a.u in A673 and 0.21 a.u in EWS502) was observed indicating the nuclear penetration of the peptide (Fig. 2A and B). The quality of the nuclear extracts was verified by performing western blots for PARP as nuclear marker and GAPDH as cytoplasmic marker. Next, a time course analysis of peptide uptake was performed at various time intervals over a period of 24 hours. The results indicated that the peptide penetration was almost instantaneous (uptake was evident at ~1 min) and TAT/NLS/EWS-PEP uptake reached a plateau by 1 hour (Fig. 2C and D).

### Inhibition of Cell growth, Colony formation and Cell Invasion in peptide treated Ewing’s Sarcoma cells

**Figure 3:**
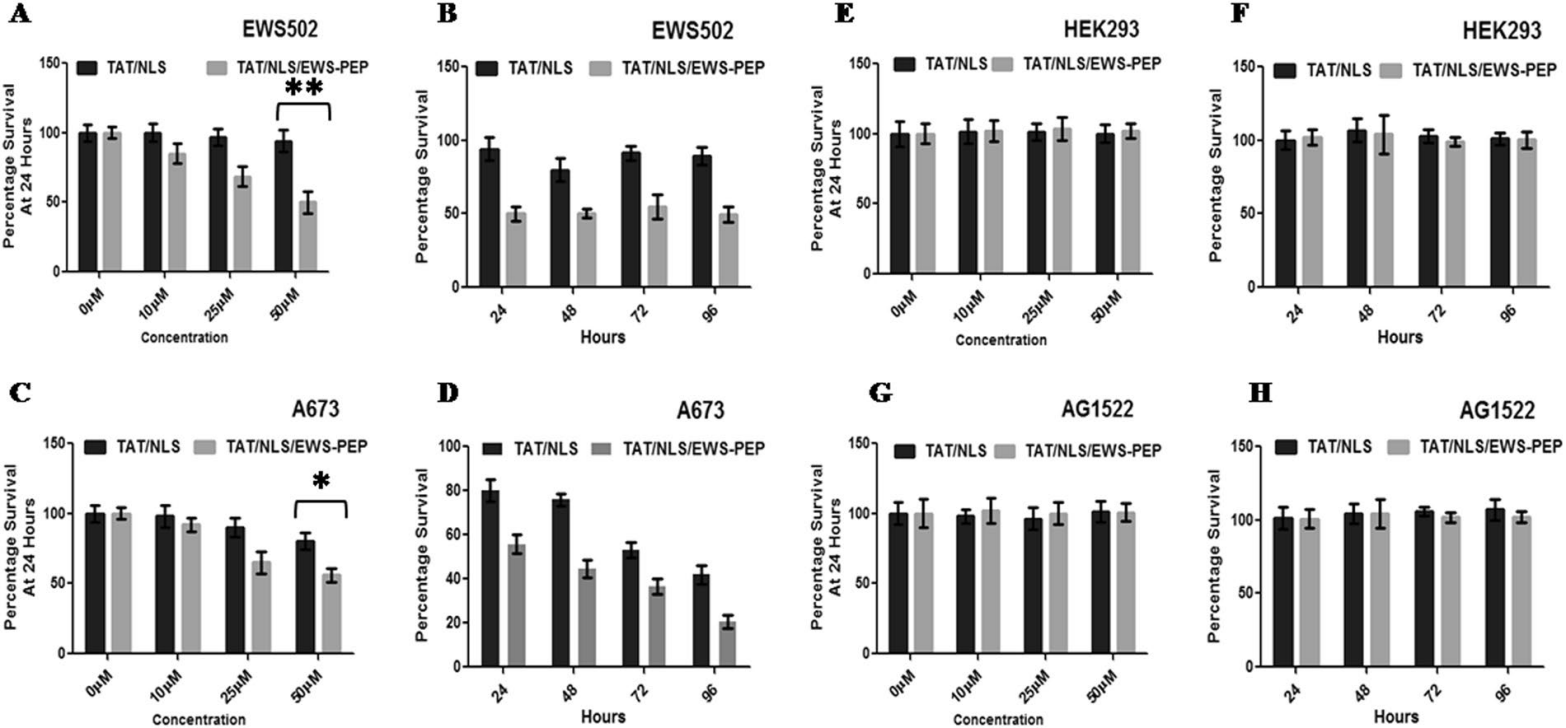
Cell proliferation analysis. Percentage survival of EWS502 in response to different concentrations of peptides (**A**) and for various time intervals (**B**). Percentage survival of A673 cells in response to different concentrations of peptides (**C**) and for various time intervals (**D**). Percentage survival of HEK293 cells in response to different concentrations of peptides (**E**) and for different time intervals. (**F**) Percentage survival of AG1522 cells in response to different concentrations of peptides (**G**) and for different time intervals (**H**). *p < 0.05, **p < 0.01.

**Figure 4:**
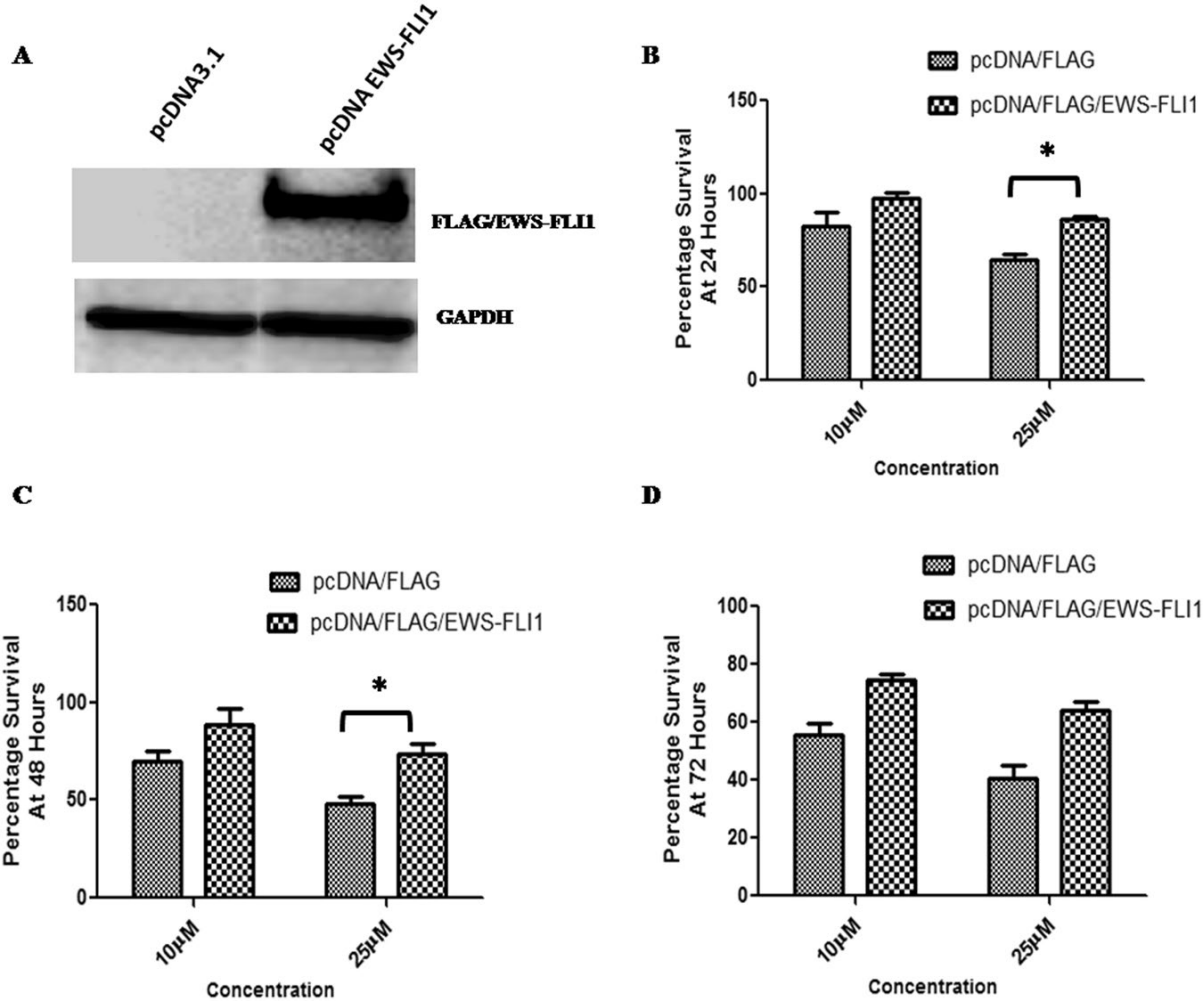
Over expression of EWS-FLI1 in A673 Cells. (**A**) Western blot confirmation of FLAG/EWS-FLI1 expression using anti-FLAG antibody. Upper panel shows the western blot probed with Anti-FLAG antibody and lower panel shows the same blot re-probed with GAPDH antibody. The cropped blots are displayed, for full blots refer supplementary information. **(B**,**C** and **D**) show percentage survival of empty vector and FLAG/EWS-FLI1 transfected cells, treated with two different concentrations of TAT/NLS/EWS-PEP for three different time points. *p < 0.05.

To understand the effects of peptide treatment on cells, unlabelled peptides were used to assess the cytotoxicity and colony formation. The results showed a dose-dependent inhibition of cell proliferation with the highest toxicity observed at 50 µM concentration relative to that of TAT-NLS alone or untreated cells in both the Ewing’s sarcoma cell line’s tested (Fig. 3A and C). Cell growth was observed over a period of 96 hours and the data indicated a decrease in cell proliferation in both EWS502 and A673 cells (Fig. 3B and D). Next, toxicity in non-tumorigenic and EWS-FLI1 translocation negative HEK293 and AG1522 cells was ascertained. The toxicity data indicated negligible toxicity in dose range (Fig. 3E and G) and time course assays (Fig. 3F and H) in comparison to Ewing’s sarcoma cells. In addition, specificity of the peptide action was assessed by transfecting full length FLAG/EWS-FLI1 into A673 cells and the effect of overexpression was determined. Initially, the over expression was confirmed by western blot using antibody against the FLAG tag (Fig. 4A). The overexpression decreased the toxicity of the TAT/NLS/EWS-PEP peptide relative to the vector alone transfected cells, indicating that the activity of the peptide at least in part occurs due to the inhibition of the EWS-FLI1 activity(Fig. 4B,C and D). Further, the colony forming ability of EWS502 cells in response to peptide treatment using soft agar assay, displayed a significant inhibition in anchorage-independent growth after TAT/NLS/EWS-PEP treatment (Supplementary Figure 1).

**Figure 5:**
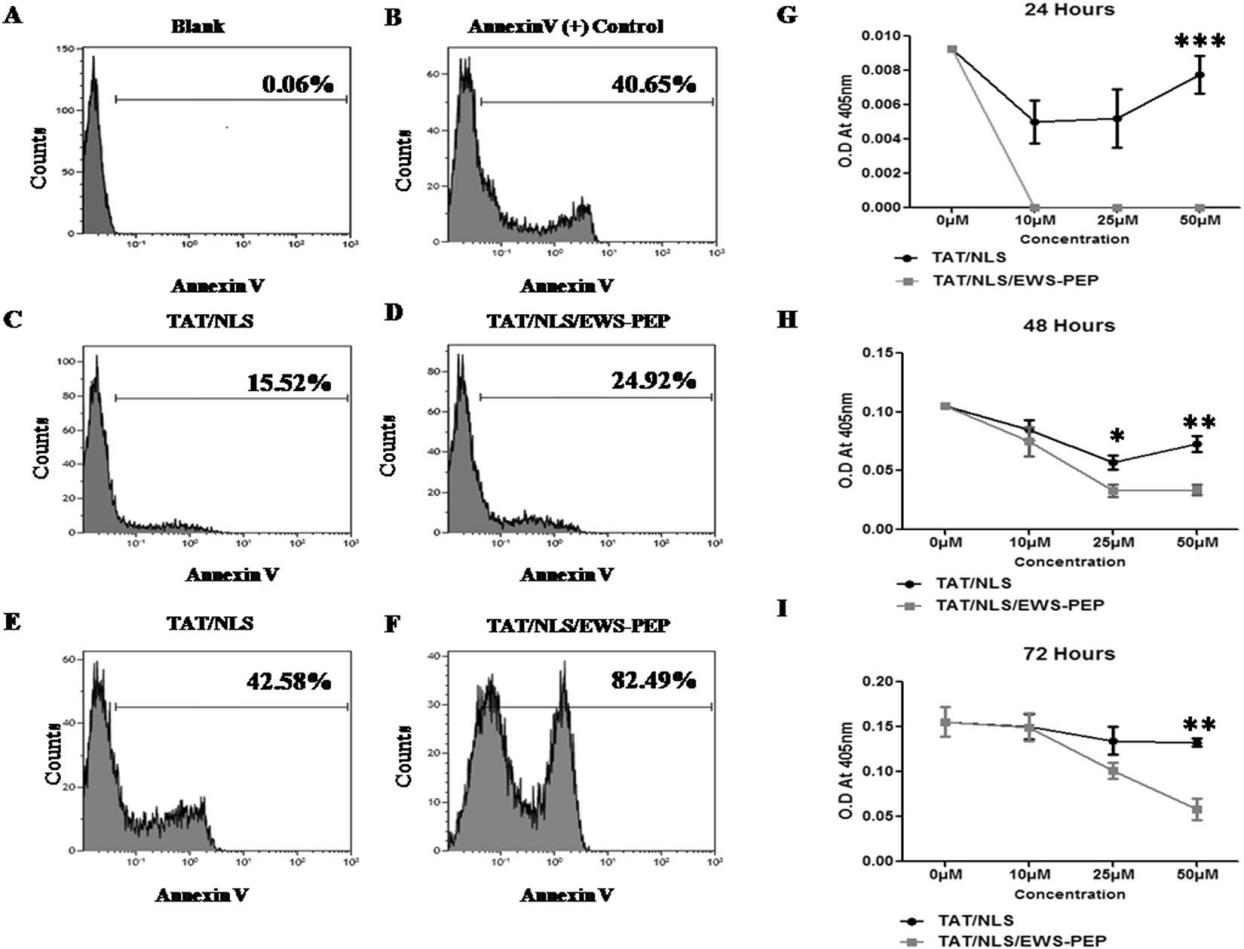
Apoptosis and BrdU incorporation analysis in A673 cells. Percentage Annexin V staining in (**A**) untreated (Blank). (**B**) Annexin V positive control. **(C** and **E)** TAT/NLS treated cells at a concentration of 50 µM and 500 µM respectively. (**D** and **F**) TAT/NLS/EWS-PEP treated cellsat a concentration of 50 µM and 500 µM respectively. **(G**,**H** and **I**) BrdU incorporation analysis of peptide treated A673 cells relative to untreated control, measured at three different time points. The line graphs represent the O.D values as a measure of BrdU incorporation. *p < 0.05, **p < 0.01.

**Figure 6:**
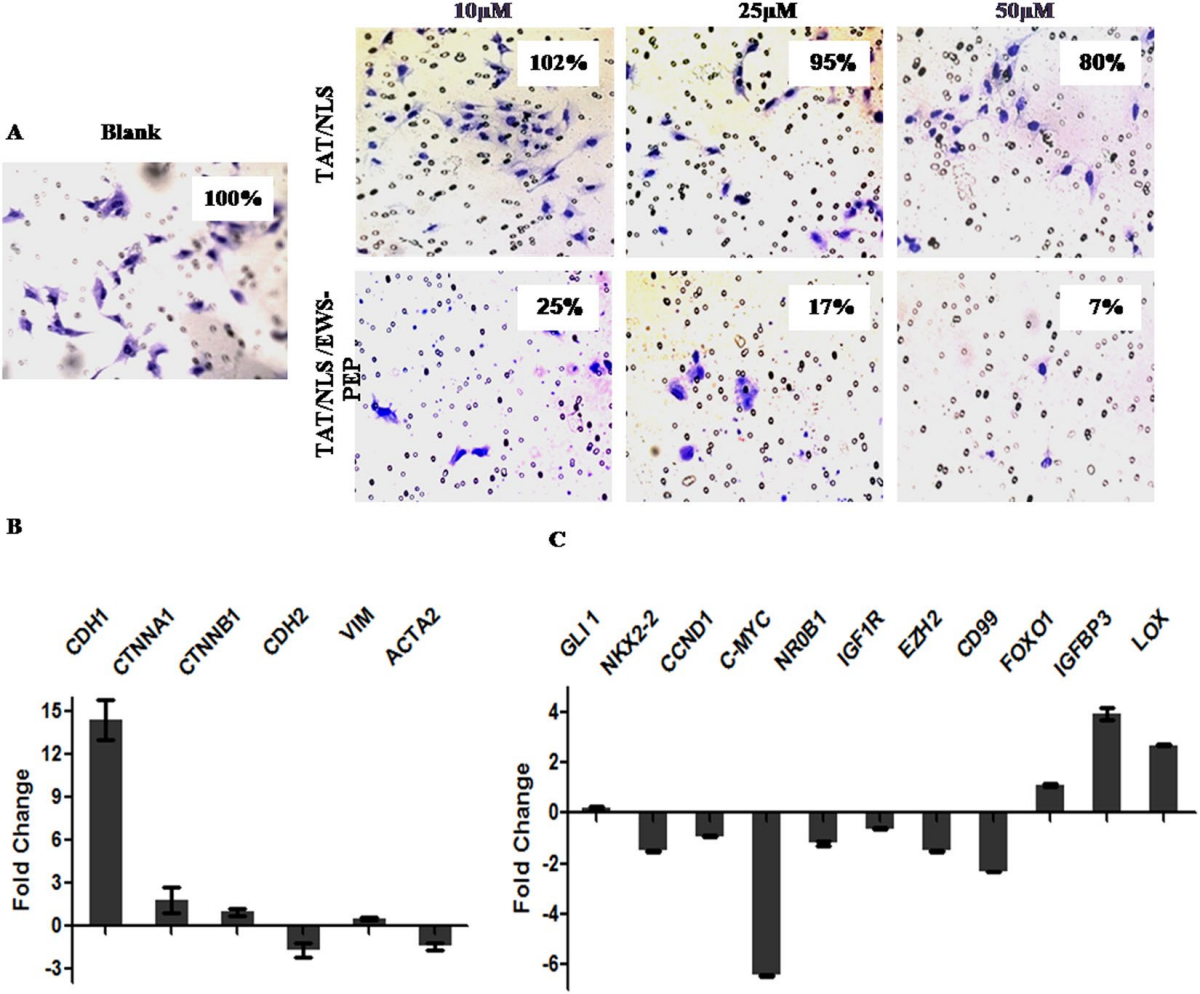
Effects of peptide on invasion and gene expression in A673 Ewing’s sarcoma cells. (**A**) Representative photographs of crystal violet stained A673 cells (either untreated or peptide treated) which invaded the matrigel layer, captured at 40× magnification. (**B**) Fold change in EMT marker genes (CDH1 – E-cadherin, CTNNA1- α catenin, CTNNB1-β catenin, CDH2 – N cadherin, VIM – vimentin, ACTA2 – α smooth muscle actin) expression levels in response to TAT/NLS/EWS-PEP treatment at 50 µM concentration. Ct values from TAT/NLS treated cells were used as reference to calculate fold change in expression and GAPDH gene expression was used for normalization. (**C**) Fold change in EWS-FLI1 target genes (GLI1 – Glioma associated oncoprotein 1, NKX2-2 – NK2 homeobox 2, CCND1-cyclin D, c-MYC, NR0B1- Nuclear Receptor subfamily 0 Group B member 1, IGF1R – Insulin like growth factor receptor 1, EZH2 – Enhancer of zeste 2 polycomb repressive complex2 subunit, FOXO1 - Fork head box protein O1, IGFBP3 – Insulin like Growth Factor Binding Protein 3 and LOX – Lysyl Oxidase expression levels in response to TAT/NLS/EWS-PEP treatment at 50 µM concentration. Ct values from TAT/NLS treated cells were used as reference to calculate fold change in expression and GAPDH gene expression was used for normalization.

Next, cell cycle analysis was performed at 48 hours following peptide treatment (50 µM) the data showed inhibition in the progression of cell cycle following TAT/NLS/EWS-PEP treatment. This was indicated by an increase in sub G1 (8.23%) and G1 (10.35%) phases and reduction in fraction of cells in S (7.06%) and G2/M (9.46%) phase’s relative to TAT-NLS treatment (Supplementary Figure 2). Since there was an increase in sub G1 population, induction of apoptosis was assessed at 50 µM peptide concentration using annexin V and heat treated cells were used as a positive control (Fig. 5B). The results revealed an increase in the apoptotic population (24.9%) in the case of TAT/NLS/EWS-PEP treatment compared to TAT/NLS treated cells (15.5%) (Fig. 5D and C). The induction of apoptosis was further increased to 82.49% in TAT/NLS/EWS-PEP treated cells and to 42.58% in TAT/NLS treated cells in presence of tenfold increased peptide concentration (Fig. 5F and E). We further assessed cell proliferation using Bromodeoxyuridine (BrdU), the assay was performed following a single pulse of peptide treatment to A673 cells at three concentrations (10 µM, 25 µM and 50 µM) and BrdU label incorporation was assessed at 24-hour intervals (Fig. 5G,H and I). The results showed a decrease in the incorporation of BrdU in TAT/NLS/EWS-PEP relative to TAT/NLS peptide treated cells with maximum difference seen in the case of 50 µM. The differences in incorporation persisted during the course of 72 hours as the cells recovered from peptide treatment confirming the dose-dependent inhibition of cell proliferation. The decrease in BrdU incorporation was consistent with a decrease in S phase fraction observed in the cell cycle analysis. Next, the effect on the invasive properties of cells following a single pulse of peptide treatment at various concentrations demonstrated a significant decrease in cell invasion in a dose-dependent manner in TAT/NLS/EWS-PEP treated cells relative to TAT/NLS treatment (73.03% reduction) (Fig. 6A). Taken together, treatment with TAT/NLS/EWS-PEP peptide induced apoptosis, inhibited growth and invasive capability of Ewing’s sarcoma cells and shows differential toxicity towards nontumorigenic cells.

### Inhibition of Epithelial to Mesenchymal transition (EMT) genes and EWS-FLI1 target gene expression in peptide treated Ewing’s Sarcoma cells

Since a decreased tumorigenic capability of cells was seen, the status of markers associated with epithelial to mesenchymal transition (E-cadherin, Alpha-Catenin, Beta-Catenin, N-cadherin, Vimentin, and Alpha smooth muscle actin) were analysed. The mRNA expression levels of these markers in response to TAT/NLS/EWS-PEP treatment with TAT/NLS treated cells acting as a control indicated a marked increase in the levels of E-cadherin (14.4 fold) in treated cells and modest reduction of N-cadherin (1.7 fold) and Alpha smooth muscle actin (ACTA2) (1.42 fold) (Fig. 6B). Next, expression levels of eight EWS-FLI1 associated target genes (GLI1, NEX2.2, CyclinD, c-MYC, NR01B, IGF1R, EZH2, and CD99) which were known to be upregulated, and three genes (FOXO1, IGFBP3, and LOX) known to be down regulated in Ewing’s sarcoma was assessed. With the exception of GLI1 all the up regulated target gene expression levels were reduced in peptide treated cells for instance, c-myc showed largest decrease in expression (fold change) (6.4) followed by CD99 (2.3), EZH2 (1.5), NEX2.2 (1.5), NR01B(1.2), CYCD(0.9) and IGF1R(0.6) and the three down regulated target genes were found to be up-regulated FOXO1 (1.12), IGFBP3 (3.9) and LOX (2.7) (Fig. 6C). These results indicated that peptide TAT/NLS/EWS-PEP can affect the transcriptional role of EWS-FLI1 protein.

### Interactions between peptide and EWS-FLI1 fusion protein

**Figure 7:**
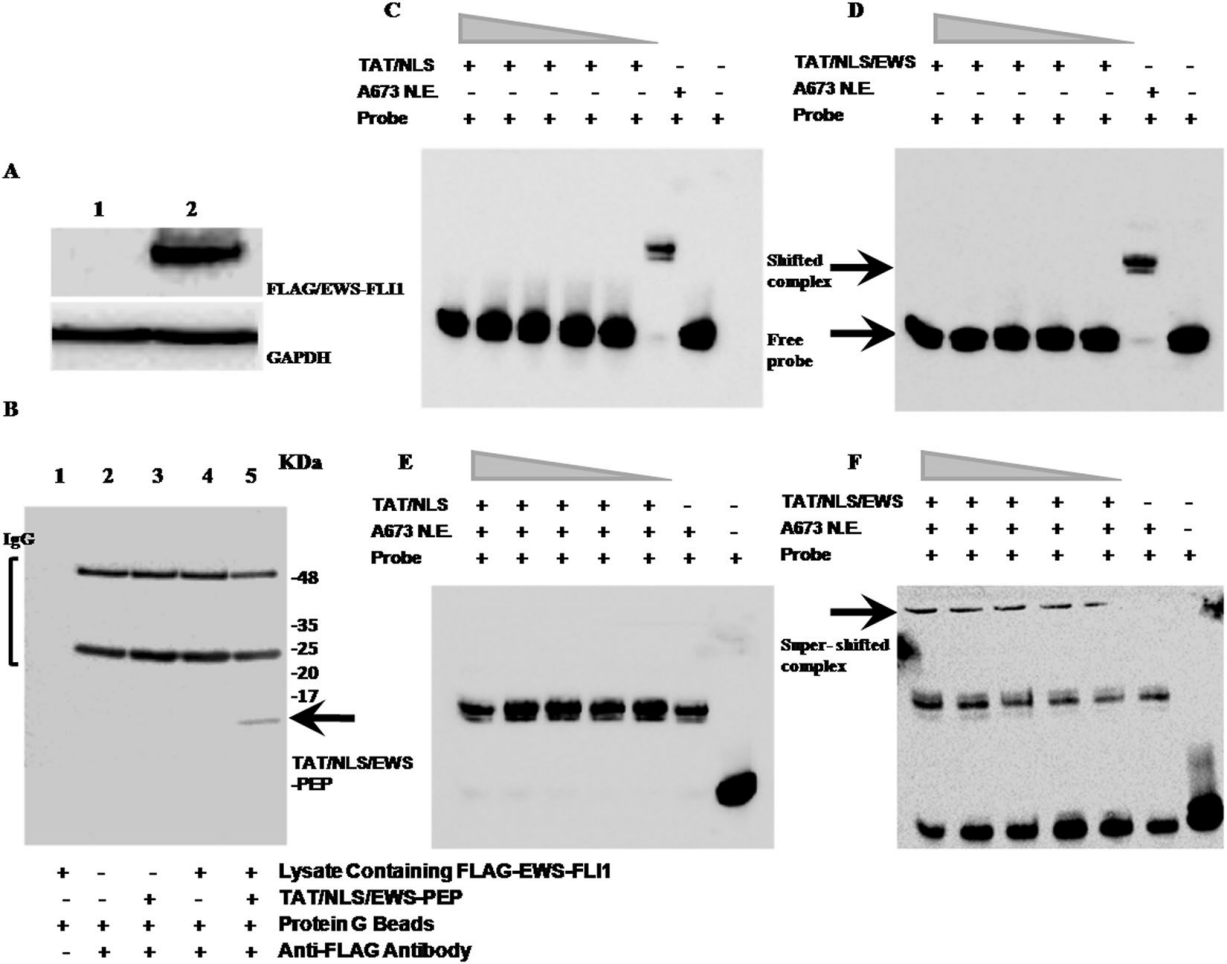
Peptide-protein and Peptide-Protein-DNA interactions of the TAT/NLS/EWS-PEP peptide. (**A**) Western blot analysis of ectopically expressed FLAG/EWS-FLI1 expression in MCF-7 cell lysates. Lanes 1: lysate from vector alone expressing cells, and 2: Lysate from FLAG/EWS-FLI1 expressing cells. Upper panel blot was probed with anti-FLAG mouse monoclonal antibody, and the lower panel shows the same blot reprobed with mouse monoclonal anti-GAPDH antibody. (**B**) Detecting TAT/NLS/EWS-PEP in FLAG immunoprecipitates (IP) using western blot: Lanes 1–4: 1: Bead control, 2: IP from MCF-7 empty vector expressing cells, 3: IP from MCF-7 empty vector expressing cells in the presence of TAT/NLS/EWS-PEP, and 4: IP from MCF-7 FLAG/EWS-FLI1 expressing cells and 5: IP from MCF-7 FLAG/EWS-FLI1 expressing cells in the presence of TAT/NLS/EWS-PEP. Full length blots are displayed in the figure. (**C**) EWS-FLI1 EMSA using 7XGGAA duplex probe. TAT/NLS peptide interaction with 7XGGAA probe. Lane 1(from right): probe alone, Lane 2: probe and A673 N.E., Lanes 3 to 7: probe with TAT/NLS at increasing concentrations (1, 2, 4, 8 and 10 μM). (**D**) EWS-FLI1 EMSA using 7XGGAA duplex probe. TAT/NLS/EWS-PEP peptide interaction with 7XGGAA probe. Lanes 1: probe alone, 2: probe and A673 N.E., Lanes 3 to 7 probe with TAT/NLS/EWS-PEP at increasing concentrations (1, 2, 4, 8 and 10 μM). (**E**) EWS-FLI1 EMSA using 7XGGAA duplex probe. TAT/NLS peptide interaction with 7xGGAA probe in presence of A673 N.E. Lane 1: probe alone, Lane 2: probe and A673 N.E, Lanes 3 to 7 mixture of probe with A673 N.E. and TAT/NLS at increasing concentrations (1, 2, 4, 8 and 10 μM). (**F**) EWS-FLI1 EMSA using 7XGGAA duplex probe. TAT/NLS/EWS-PEP peptide interaction with 7XGGAA probe in presence of A673 N.E., Lane 1: probe alone, Lane 2: probe and A673 N.E., Lane 3 to 7 mixture of probe with A673 N.E., andTAT/NLS/EWS-PEP at increasing concentrations (1, 2, 4, 8 and 10 μM).

Since gene expression analysis showed that the transcriptional activity of the EWS-FLI1 could be affected by TAT/NLS/EWS- PEP, we wanted to assess whether this would probably involve interaction of the peptide with the wild-type protein. To assess for the peptide-protein interaction, full-length FLAG-tagged EWS-FLI1 was expressed in MCF-7 breast cancer cells used as an expression host (Fig. 7A). The choice of a mammalian cell culture system for the production of recombinant EWS-FLI1 was made since our previous work had shown that the expression of recombinant EWS-FLI-1 (for interaction studies) with various tags (GST, 6XHis, Thioredoxin) in bacterial cells resulted in the protein being present in the inclusion bodies and could not be purified in the native form (8). Following immunoprecipitation with anti-FLAG antibody coated magnetic beads in the presence or absence of the peptide, the pulldown complex was analysed for the presence of the peptide using anti-TAT antibody. The results from the immunoblot analysis indicated the presence of peptide in FLAG/EWS-FLI1 immunoprecipitates incubated with the TAT/NLS/EWS-PEP whereas the immunoprecipitates from the vector alone expressing cell lysate, or a combination of vector alone expressing cell lysate and TAT/NLS/EWS-PEP, or EWS-FLI1 expressing cell lysate did not show positivity for the peptide (Fig. 7B). The experiment was repeated once more and the interaction was confirmed.

EWS-FLI1 is known to function as transcription factor and bind to GGAA microsatellite repeat sequences^10^; therefore we further assessed the protein-peptide interaction in the presence of GGAA repeat sequences. Electrophoretic Mobility Shift Assay (EMSA) was performed using nuclear extract (N.E) from A673 cells. The results showed that nuclear extract from A673 cells formed complex with the 7X GGAA probe and the specificity of complex was confirmed by competition with the unlabelled probe (Supplementary Figure 3). Next, we tested whether the peptides themselves were capable of interacting with the probe, in the case of both TAT-NLS and TAT/NLS/EWS-PEP no probe shift was detected at various concentrations tested (1, 2, 4, 8 and 10 μM), whereas the nuclear extract used as positive control showed complex formation (Fig. 7C and D). Finally, the effect of the peptide on DNA-protein complex formation was ascertained. In the case of TAT-NLS peptide, the presence did not affect the complex formation since there was no further shift in complex mobility relative to that formed in the presence of nuclear extract alone (Fig. 7E). However, with the TAT/NLS/EWS-PEP, we found a distinct super shift of the complex in the presence of the peptide (Fig. 7F). The results from peptide interaction studies indicate that the peptide is capable of interacting with EWS-FLI1 protein and this interaction can occur in the presence of the GGAA microsatellite repeat sequences.

### Identification of peptide-protein interactions

Next, we analysed for potential peptide-protein interactions in Ewing’s sarcoma cells which could provide clues to understand its activity. Since lysine residues were present in the TAT/NLS sequence, the peptides were subjected to biotinylation and confirmed in ELISA using streptavidin –HRP conjugate. Both the labelled peptides showed reactivity, whereas the free biotin on account of poor immobilization on the surface showed levels close to that of background (Supplementary Figure 4A). Next, the saturating concentration of the peptides for the streptavidin beads was approximately assessed using various amounts of the peptide against a fixed volume of beads. Results from the analysis showed that at 1 µg of input, the peptide was detectable in the flow through indicating saturation of the streptavidin beads for both TAT/NLS and TAT/NLS/EWS-PEP (Supplementary Figure 4B and C). Pull-down assays were performed using nuclear extract from A673 cells (Supplementary Figure 5) since previously it was shown that EWS-FLI1 protein interacts with nuclear proteins and is involved in regulation of transcription^11^. The fraction of proteins bound to the streptavidin beads (control) were excluded from the proteins bound to TAT/NLS and TAT/NLS/EWS-PEP peptide fractions. The resultant list was assessed for proteins exclusively present in either of the peptide pull-downs, in the TAT/NLS/EWS-PEP list 12 proteins, TAT/NLS 12 proteins and 3 proteins common to both peptide pull-downs were identified (Supplementary Table 2). The TAT/NLS/EWS-PEP pull down list included proteins previously identified in EWS-FLI1 interacting complexes, for instance, TBB3, NPM, EF1-D, ENPL, RL17, HNRPC, TBB5 (Table 1). Taken together the streptavidin-based affinity pull down coupled with nLC/MS/MS analysis indicate that TAT/NLS/EWS-PEP can interact with proteins present in EWS-FLI1 protein complexes.

**Table 1:** List of proteins identified in TAT/NLS/EWS-PEP pull down and is known to be potential interacting partners of EWS-FLI1.

Proteins Identified	Unique Peptides
Tubulin beta chain (TBB5)	K.MAVTFIGNSTAIQELFKR.I
Tubulin beta-3 chain (TBB3)	K.VAVCDIPPRGLK.M
Nucleophosmin (NPM1)	R.MTDQEAIQDLWQWRK.S
Elongation factor 1-delta (EF1D)	R.IASLEVENQSLR.G
Endoplasmin (ENPL)	K.FAFQAEVNR.M
	K.SILFVPTSAPR.G
60S ribosomal protein L17 (RL17)	K.SAEFLLHMLK.N
	R.ETAQAIKGMHIR.K
Heterogeneous nuclear ribonucleoproteins C1/C2 (HNRPC)	R.VFIGNLNTLVVK.K
	K.GFAFVQYVNER.N

## Discussion

Peptides can be utilized in a number of different ways in treating cancer. This includes using peptides directly as drugs (eg: Angiogenesis inhibitors), tumour targeting agents that carry cytotoxic drugs and radionuclides (targeted chemotherapy and radiation therapy), hormones and vaccines^12^. Anti-cancer peptides (ACP) have attracted widespread attention as a new class of anti-cancer drug candidate due to their high activity and unique mechanism of targeting cells. Although the exact mechanism of action of ACP is unclear, most ACPs appear to affect multiple cellular processes, including necrosis, apoptosis and gene expression to kill cancer cells^13^. Compared with the traditional cancer treatments such as chemotherapy or radioactive treatment, peptides with high specificity against cancer cells may present a way of killing cancer cells while protecting normal cells^14^.

Targeting EWS-FLI1 is potentially efficacious since there is evidence for a direct role in tumorigenesis through an altered transcriptional program executed by the chimeric protein in tumour cells; in addition the translocation occurs in the background of low mutation rate^15^ further affirming its role in the aetiology of Ewing’s sarcoma. In order to better understand the role of the junction region (a.a. 251–280) sequences, which we had previously shown to inhibit the tumorigenic properties of Ewing’s sarcoma cells^8^, the amino acids corresponding to this region were introduced into Ewing’s sarcoma cells and its effects were analysed. Since the peptide comprising of the junction region amino acids (a.a. 251–280) did not by itself have cell penetrating properties, a well-studied soluble cell penetrating peptide (CPP) sequence from HIV-Tat protein^16, 17^ was incorporated into the sequence. Based on the fact that EWS-FLI1 functions as transcription factor and is located in the nucleus, we hypothesized that targeting the peptide to the nucleus could increase its effectiveness and the peptide can function as bait depriving the EWS-FLI1 protein of interacting protein partners or prevent its DNA binding ability and therefore compromise its activity. To this end we introduced NLS sequences in addition to CPP sequence to maximize targeting of the peptide to the nucleus of cells. As predicted the TAT-NLS tag enabled both cell penetration and nuclear localization in treated cells. Since, previous studies^18, 19^ had shown that TAT peptide can induce certain amount of cytotoxicity in treated cells; we used the TAT-NLS peptide as control to delineate the effects of the tag from that of TAT/NLS/EWS-PEP. However, functional effects of different amino acids in the fusion region of EWS-FLI1 were not determined hence it was not optimal to design effective mutant form of the target peptide which could possess a contraindicating activity. When the peptide was introduced into Ewing’s sarcoma cells we observed an inhibition of cell proliferation, colony formation, cell cycle and invasive properties. Interestingly, over expressing EWS-FLI1 in A673 cells significantly reduced the toxicity associated with TAT/NLS/EWS-PEP treatment compared to the empty vector transfected cells, indicating a degree of potential target specificity to peptide action. In addition, among the EMT markers there was significant increase in levels of E-cadherin expression and modest decrease in the levels of mesenchymal markers N-cadherin and ACTA2 which could result as a consequence of the inhibition of tumorigenic properties.

EWS-FLI1 as a transcription factor can alter the gene expression patterns by both gene activation and repression and a number of genes have been reported to be its targets. For instance NR01B, TGFβRII, cyclinD1, c-Myc, IGFBP3, PTPL1, cyclinE, caveolin1, LOX and FOXO1^20–28^. When assessed for effects on gene expression levels of the target genes we found that levels were reversed relative to the TAT/NLS treated cells indicating a potential functional inhibition of EWS-FLI1 transcriptional activity. Since the presence of peptide was affecting the transcriptional activity of EWS-FLI1 we tested for a potential interaction with the EWS-FLI1 protein. To this end co-immunoprecipitation (Co-IP) experiments using ectopically expressed EWS-FLI1 protein were performed and the result showed that peptide is capable of interacting with the EWS-FLI1 protein complex. Since EWS-FLI1 is known to bind to GGAA microsatellite repeat sequences as a homodimer^29^, we further assessed whether the peptide was capable of interacting with the protein-GGAA sequence complex using EMSA. Our results demonstrated that the peptide interacts with the DNA protein complex and plausibly through its interaction affect the transcriptional activity of the EWS-FLI1 protein.

Previous reports have indicated that EWS-FLI1 is involved in several protein-protein interactions and plays a significant role in the pathways encompassing these interactions^30^. Taking note of the fact that lysine residues were restricted to the TAT/NLS sequences, pull-down experiments were performed using the biotin labelled peptides. Analysis of the protein fraction using nLC/MS/MS showed that proteins uniquely present in the TAT/NLS/EWS-PEP peptide pull-downs were previously reported to potentially interact with EWS-FLI1 protein. For instance, one of the proteins identified in the pull-downs was Nucleophosmin (NPM1). NPM is a nuclear protein widely over-expressed in various cancers^31^, including Ewing’s sarcoma^32^. NPM over-expression was frequently found in haematological malignancies associated with chromosomal translocation^33^. NPM is found to be more abundant in tumour and growing cells than in normal resting cells^34^ and it’s over expression in NIH3T3 cells resulted in malignant transformation^35^. NPM expression is down regulated in cells undergoing differentiation or apoptosis^36, 37^ and it’s over expression leads to inhibition of apoptotic cell death^38^. At present it is not known how EWS-FLI1 influences the activity of Nucleophosmin and vice-versa. Based on our data we could potentially hypothesize that the binding of the peptide could disrupt the interaction of EWS-FLI1 with Nucleophosmin or peptide’s interactions with Nucleophosmin on its own effect the inhibition of tumorigenic properties of Ewing’s sarcoma cells.

In summary, we have identified a soluble peptide comprising of amino acids from the fusion region of EWS-FLI1 gene which can inhibit the tumorigenic properties of Ewing’s sarcoma cells *in-vitro*, and interact with EWS-FLI1, EWS-FLI1- DNA complex and with proteins present in EWS-FLI1 complexes. As a consequence we find scope for design optimization/modifications to the peptide which can help disrupt the EWS-FLI1-DNA interactions or protein-protein interactions that can potentially compromise the transcriptional activity of the chimeric protein.

## Materials and Methods

### Peptides

Three different types of peptide were used in the study (Supplementary Table 1). EWS-PEP, TAT-NLS peptide, and a peptide composed of a combination of TAT-NLS and EWS-PEP(TAT/NLS/EWS-PEP). The FITC labelled peptides were obtained from GenScript (USA) and unlabelled peptides were obtained from ThermoFisher (USA).

### Cell lines, Cloning and Peptide transfection

Ewing’s sarcoma cell lines - EWS502 and A673, Human embryonic kidney derived HEK293 cells and AG1522 normal human skin fibroblast cells were maintained in cell culture medium as previously described^8^. Sequence verified full-length cDNA corresponding to Type 1 EWS-FLI1 fusion gene was expressed using pcDNA3.1/FLAG mammalian expression vector. Peptide treatment was performed following serum starvation of cells at 37 °C for 30 minutes, cells were counted and a requisite number of viable cells was mixed with peptide and incubated at 37 °C for 1 hour. MCF-7 breast cancer cell line was grown in DMEM supplemented with 10% Fetal Bovine Serum. 5 μg of pcDNA 3.1/FLAG EWS-FLI1 and pcDNA 3.1/FLAG plasmid alone were transfected separately into MCF-7 using X-treme^TM^ GENE 9 (Roche, Indianapolis, USA) transfection reagent as per the manufacturer’s instructions. The transfected cell line was selected in 100 ug/ml hygromycin (Invitrogen) and maintained in the presence of hygromycin. Single clone of transfected cells was isolated by serial dilution, and the expression of FLAG tagged EWS-FLI1 was confirmed.

### Fluorescence Activated Cell Sorting (FACS) analysis and Fluorescence imaging

FACS analysis was performed to assess the efficiency of the peptide uptake in A673 and EWS502 cells. After incubation with FITC labelled peptides, cells were washed in phosphate-buffered saline (PBS) twice and analysed in Mo-Flo™ XDP (Beckman coulter, USA). A673 and EWS502 cells were washed in serum-free medium and incubated with FITC labelled peptides for 1 hour at 37 °C, fixed in 3% formaldehyde and stained with 0.1% DAPI followed by imaging (Sigma-Aldrich, USA). For measuring intracellular peptide uptake, A673 cells were lysed and the fluorescence was quantified using a Fluoroskan Ascent^TM^ microplate Fluorometer (Thermo Scientific).

### Time-course uptake of Peptides

To track the uptake of peptides in a time-dependent manner, approximately 0.05 × 10^6^ A673 and EWS502 cells were seeded per well in a 24-well plate and cultured using complete growth medium. 24 Hours post seeding, the cells were washed in serum free medium twice and incubated with 10 μM concentration of FITC labeled peptides (TAT/NLS, TAT/NLS/EWS-PEP) for various time points (1 min, 5 min, 15 min, 30 min, 1Hour, 6Hours and 24 Hours). Following the incubation period, the cells were lysed in Tris buffer (20 mM Tris pH 8, 137 mM NaCl, 1% NP-40 and 2 mM EDTA) and centrifuged. The fluorescence intensity of the supernatant was measured using Fluoroskan Ascent^TM^ Microplate Fluorometer (Thermo Scientific, Massachusetts, USA) was normalized to the protein concentration.

### Estimating the nuclear localization of peptides

Both A673 and EWS502 cells were grown in a 100 mm dish for 24 hours and treated with FITC peptides for 1 hour in serum free medium. Preparation of nuclear extract was carried out using NE-PER nuclear and cytoplasmic extraction kit according to the manufacturer’s protocol (Thermo Scientific, USA). The fluorescence intensity was measured using Fluoroskan Ascent^TM^ Microplate Fluorometer (Thermo Scientific, USA).

### Cell proliferation and Colony formation assays

3-(4,5-dimethyl-2yl)-5-(3-carboxymethoxyphenyl)-2-(-4-sulfophenyl)-2H-tetrazolium, inner salt (MTS) proliferation assay was carried out according to the manufacturer’s protocol (Promega, USA). The soft agar colony formation assay in EWS502 cells was performed as previously described^39, 40^.

### Cell cycle and BrdU incorporation assay

For cell cycle analysis, the cells were harvested 48 hours after peptide treatment and fixed in ethanol. The fixed cells were washed twice in PBS and incubated in PBS containing RNaseA (Sigma-Aldrich, USA) at 37 °C for 30 min. After staining with 1 mg/ml PI (Invitrogen, USA) in 0.1% Triton-X-100, the cells were analysed in Mo-Flo™ XDP (Beckman coulter, USA). The cell cycle distribution was analyzed using summit software (Maumelle, USA). BrdU cell proliferation assay was performed using BrdU labelling and detection kit III (Roche, USA), after incubating A673 cells (both untreated and peptide treated) with 10 μM concentration of BrdU for 18 hours as per the manufacturer’s protocol.

### Annexin V staining

Peptide treatment was performed for three hours and annexin-v staining of A673 cells was carried out using FITC Annexin V Dead cell apoptosis kit (Molecular probes, USA) as per manufacturer’s instructions. Apoptosis was induced in A673 cells by heating at 50 °C for 20 min and was used as positive control for Annexin V binding whereas unstained cells served as blank to set the gating parameters.

### Matrigel invasion assay

Untreated and peptide treated A673 cells (2 × 10^4^ per well), were resuspended in 0.5 ml of 1% FBS containing DMEM and added to the matrigel coated inserts, 6.5 mm wide/8 μM pore size (Costar, Corning, USA), which were then placed into the wells of a 24-well companion plate, containing 1 ml of 10% DMEM as chemo attractant. The plate was incubated at 37 °C for 48 hours. Cells that migrated to the lower surface of the membrane were fixed in 3.7% paraformaldehyde and stained with 0.05% crystal violet.

### Real-time quantitative Polymerase Chain Reaction (RT-PCR)

For gene expression analysis, cDNA obtained from untreated and peptide treated A673 cells was used. The assay was performed as previously described using the primers (Supplementary Table 3) and conditions^8^. The relative fold difference in expression of the target gene between the control and peptide treated cells were analyzed with 2^ΔΔCT^ method.

### EMSA

EMSA for EWS-FLI1 was done using Light Shift® Chemiluminescent EMSA kit (Pierce Biotechnology, USA). Approximately 10 μg of A673 nuclear extract was incubated with different concentrations of TAT-NLS and TAT-NLS-EWS for 1 hour at 4 °C. 7X GGAA probe specific for EWS-FLI1 was synthesized and 5′ end labelled with biotin. Binding reaction was set up with the following components - 2 μl of 10X binding buffer, 2.5% glycerol, 5 mM MgCl_2_, 1 μg of poly (dI.dC), 0.05% NP-40, 4pM of unlabelled 7X GGAA probe, 10 μg of A673 nuclear extract (with or without peptide) and 20fM of biotin labelled 7X GGAA probe. The reaction mixture was incubated at room temperature for 20 min. The complexes were resolved in a 5% polyacrylamide gel in 0.5X TBE, transferred to nylon membrane and developed using Chemiluminescent nucleic acid detection module (Pierce Biotechnology, USA).

### Cloning and expression of EWS-FLI1 in Ewing’s sarcoma cells

The sequence verified EWS-FLI1 Type 1 fusion gene was cloned into pcDNA3.1/FLAG vector at EcoRV and Xho I restriction sites as previously described^8^. A673 Ewing’s sarcoma cell line was grown in DMEM supplemented with 10% Fetal Bovine Serum. 2 μg of pcDNA 3.1/FLAG EWS-FLI1 and pcDNA 3.1/FLAG plasmid alone were transfected separately into A673 cell line (harbouring EWS-FLI1 fusion gene) using X-treme^TM^ GENE 9 (Roche, Indianapolis, USA) transfection reagent as per the manufacturer’s instructions. 48 hours post transfection, the cells were lysed in RIPA buffer and the expression of FLAG EWS-FLI1 was confirmed by western blot using Anti-FLAG antibody procured from Cell Signalling Technologies® (Danvers, MA, USA). The proliferation of the transfected cells, with or without peptide treatment was determined using MTS reagent.

### Immunoprecipitation and western blot

MCF-7 breast cancer cells were stably transfected with FLAG EWS-FLI1 expressing pcDNA 3.1 construct and empty pcDNA3.1 vector as control. The expression of EWS-FLI1 was confirmed by western blotting against FLAG using anti-FLAG antibody (Sigma®, Clone M2). Immunoprecipitation was performed using 1.5 mg of the lysates, and 10 μg of the peptides - TAT-NLS and TAT-NLS-EWS. Western blotting for peptide detection was performed by running the complex in 12% SDS-PAGE gel, electrotransferred onto 0.2 μM pore size polyvinylidene difluoride membrane (NovexLife technologies, USA) and probed using mouse anti-HIV-TAT antibody (Thermo scientific®, USA).

### Biotin Labelling of Peptides

The peptides (500 µg, 5 µg/µl) were labelled with biotin using Proton™ biotin labelling kit (Vector Laboratories, USA) as per manufacturer’s protocol. Unreacted biotin was removed by dialysis using Slide-A-Lyzer™ mini dialysis devices, 2KDa cut off (Thermo scientific, USA).

### Streptavidin-Biotin-Peptide pull down

1 ml of A673 nuclear extract (1 µg/µl) was precleared using 30 µl of streptavidin agarose beads (Vector Laboratories, USA) by incubating for 2 hours at 4 °C in a rotator. 50 µl of agarose streptavidin beads were coated with 1 µg of biotinylated peptides TAT/NLS and TAT/NLS/EWS-PEP in 0.1% NP-40 PBS for 2 hours at 4 °C. The coated beads were mixed with precleared A673 nuclear extract in a modified RIPA buffer (50 m M Tris-HCl pH 7.5, 150 mM NaCl, 1% NP-40, 1 m M EDTA, 1 m M EGTA, 0.1% SDS, Protease inhibitor cocktail (Roche, Switzerland), and 0.5% sodium deoxycholate) overnight at 4 °C. The beads were washed in 50 mM Ammonium bicarbonate (Ambic) buffer and treated with DTT followed by 15 mM Iodoacetamide. 50 ng of sequencing grade trypsin (Roche, Basel, Switzerland) was added to the beads and incubated at 37 °C overnight for digestion. The samples were desalted using C18 zip-tips (Millipore, USA) and eluted in 10 µl of 60% Acetonitrile and 40% formic acid, dried and finally the peptides were dissolved in 0.1% formic acid water.

### Nano Liquid Chromatography – Mass Spectrometry (nLC-MS/MS) analysis of Peptide mixture

To the desalted peptides nLC-MS/MS was performed using Easy nLC II (Bruker Daltoniks, Germany) with C18 matrix packed precolumns (NanoEase Trap column, 100 µM ID, 5 µM) and analytical columns (NanoACUITY, 150mm, 75 µuM ID, 3.5 µM, C18 nanocolumn) supplied by Waters (Milford, USA) designated easy nano-LC columns. The mobile phase consisted of (A) 0.05% v/v formic acid in water (LC-MS CHORMASOLV^®^, Sigma-Aldrich, USA) and (B) 0.05% v/v formic acid, 95% v/v ACN in water (LC-MS CHORMASOLV^®^, Sigma-Aldrich, USA) nano LC was performed using Easy nano LC system supplied from Bruker. The mass spectrometry analysis was performed using ESI-Trap mass spectrometer (amaZon ETD ion trap, BrukerDaltonics, Germany) equipped with ESI source (Captive Spray^®^, BrukerDaltonics, Germany). The parameters used for measurements were capillary voltage 1500 V; dry gas 3.0 L/min; dry temperature 150 °C; Nebulizer 10 psi; and the scan mode was set to UltraScan and measurements were performed in the positive ion mode. The mass tolerance for the protein search was set at 1 Da for precursor mass selection (MS) and 0.5 Da for fragment mass (MS/MS), two missed cleavage sites were allowed and the number of hits was set to auto. The criteria used for listing out proteins was based on the cut-off score set by Mascot algorithm (Protein Score) combined with a minimum of one or two unique peptides matching the protein sequence.

### Statistics

The Standard deviation was calculated from replicate experiment values and results are expressed as means ± SD. For comparisons between two groups, P values were calculated with paired, two-tailed Student’s t-tests.

## Electronic supplementary material

Supplementary Information
