# Supplementary material for: A polypeptide from the junction region sequence of EWS-FLI1 inhibits Ewing’s sarcoma cells, interacts with the EWS-FLI1 and partner proteins
Source: Sci Rep. 2017 Aug 3;7:7172. doi: 10.1038/s41598-017-07482-4 (PMC5543137; doi:10.1038/s41598-017-07482-4)

**A polypeptide from the junction region sequence of EWS-FLI1 inhibits Ewing’s sarcoma cells, interacts with the EWS-FLI1 and partner proteins.**

Krishna Priya Thangaretnam^1^, Gopal Gopisetty^1,2^, Priya Ramanathan^1^, Thangarajan Rajkumar^1*^.

Krishna PriyaThangaretnam: priyabiochem6@gmail.com

Gopal Gopisetty: gopisettygopal@yahoo.com

Priya Ramanathan: priya.serkin@gmail.com

1- Department of Molecular Oncology, Cancer Institute (WIA), Chennai, India. 2- Co-Corresponding Author *- Corresponding Author.

Address for correspondence:

Dr. Thangarajan Rajkumar,

Prof. and Head,

Department of Molecular Oncology,

Cancer Institute (WIA),

38, Sardar Patel Road, Chennai 600 036, Tamil Nadu,

India.

Tel: +91 4422350340.

Fax: +91 4424912085.

E-mail: drtrajkumar@gmail.com

# Supplementary Figures


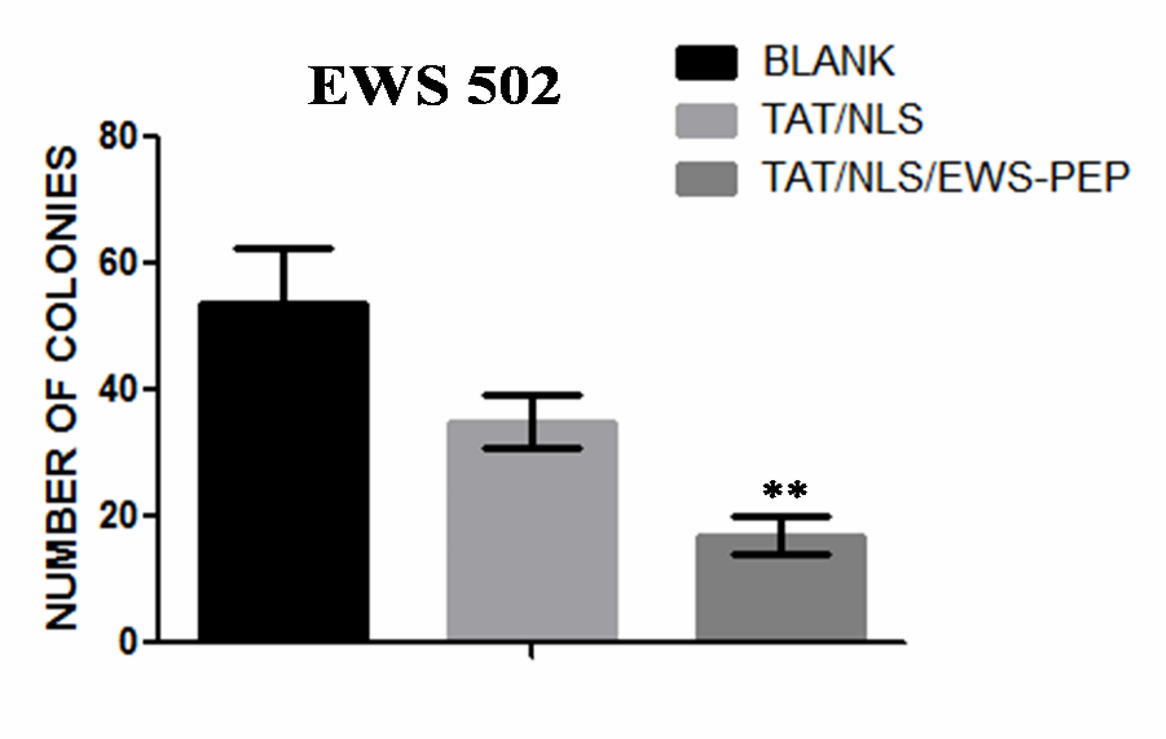


**Supplementary figure 1**: Colony formation of peptide treated EWS502 cells in soft agar. After a period of 21 days following peptide treatment, the colonies were counted in 10 randomly chosen microscope fields. **p‹0.01.


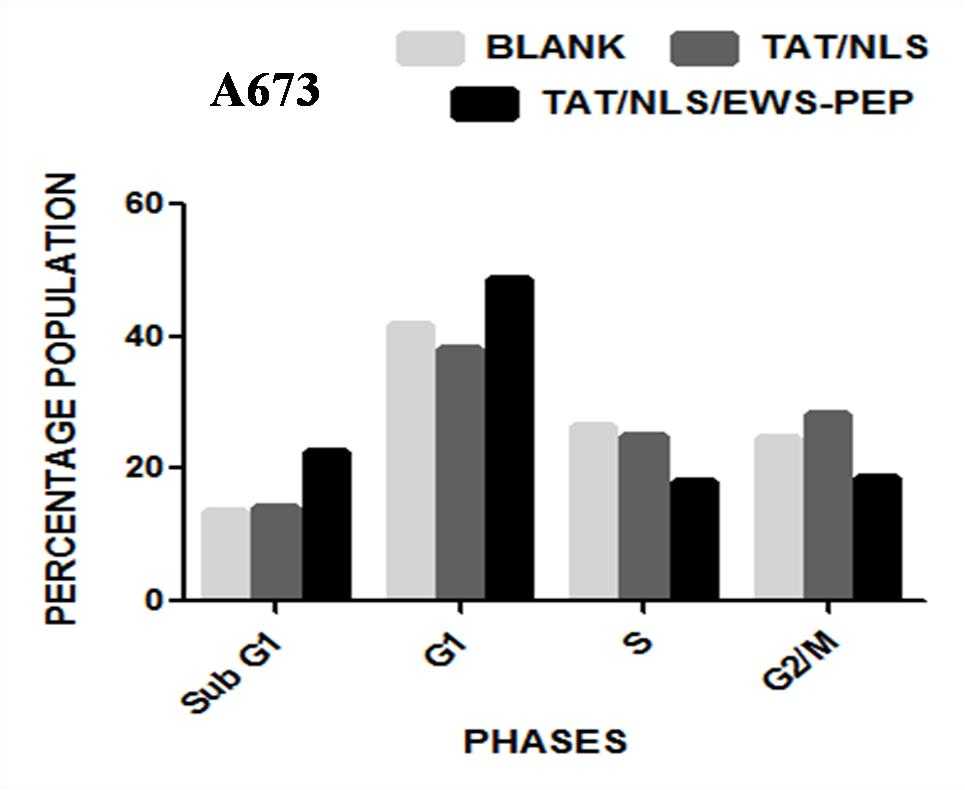


**Supplementary figure 2**: Distribution of A673 cells in various phases of the cell cycle in untreated (Blank) or 48 hours after treatment with TAT/NLS or TAT/NLS/EWS-PEP at a concentration of 50µM.


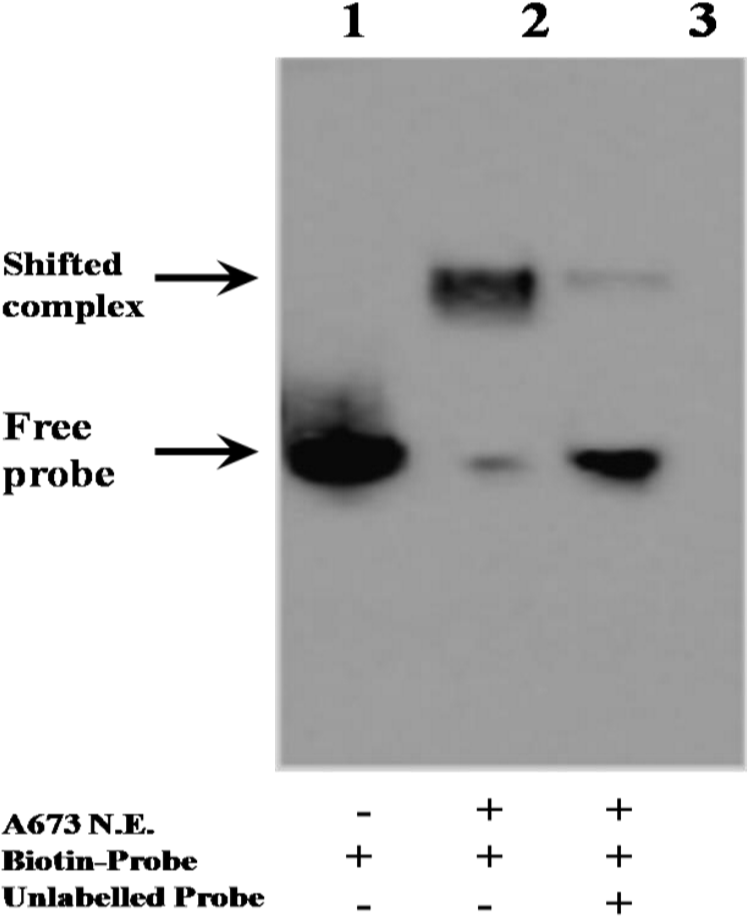


**Supplementary figure 3**: EWS-FLI1 EMSA using 7XGGAA probe. Lane 1: probe alone, Lane 2: mixture of A673 N.E. and probe, Lane 3: mixture of unlabelled probe (200 fold molar excess than labelled probe), labelled probe and A673 N.E.


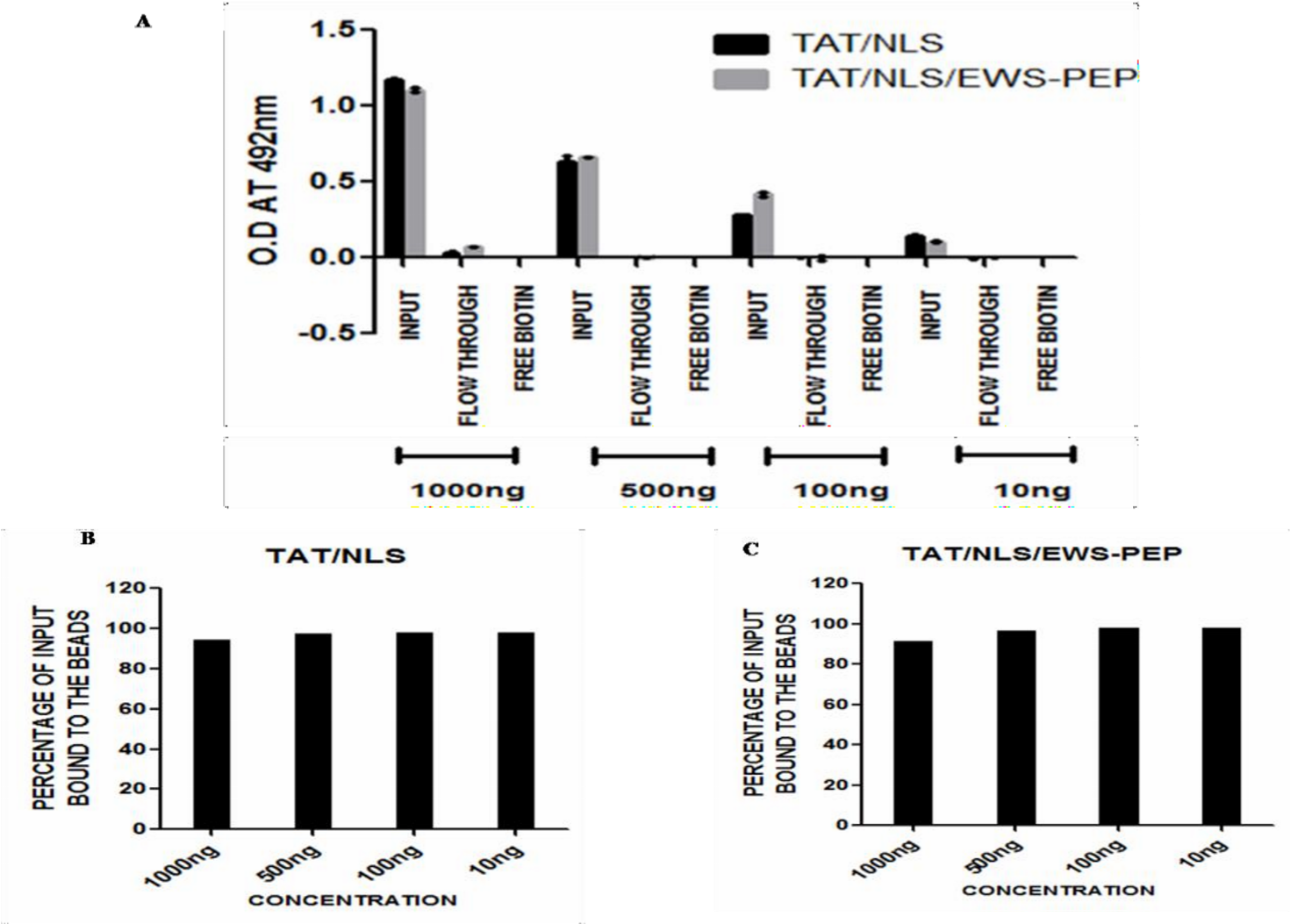


# Supplementary Figure 4

**(A)**: Biotinylation of peptides. Supplementary Figure 4A: ELISA O.D values for different concentrations of biotinylated peptides - TAT/NLS and TAT/NLS/EWS, Input and flow through along with free biotin

**(B)**: Binding of biotinylated TAT/NLS to strepatavidin beads (50 µl). The ELISA plate was coated with input peptide and the corresponding flow through following incubation with beads. The percentage of input bound to the beads was estimated as a fraction of the peptide in flow through compared to the input.

**(C)**: Binding of biotinylated TAT/NLS/EWS-PEP to strepatavidin beads (50 µl). The ELISA plate was coated with input peptide and the corresponding flow through following incubation with beads. The percentage of input bound to the beads was estimated as a fraction of the peptide in flow through compared to the input.


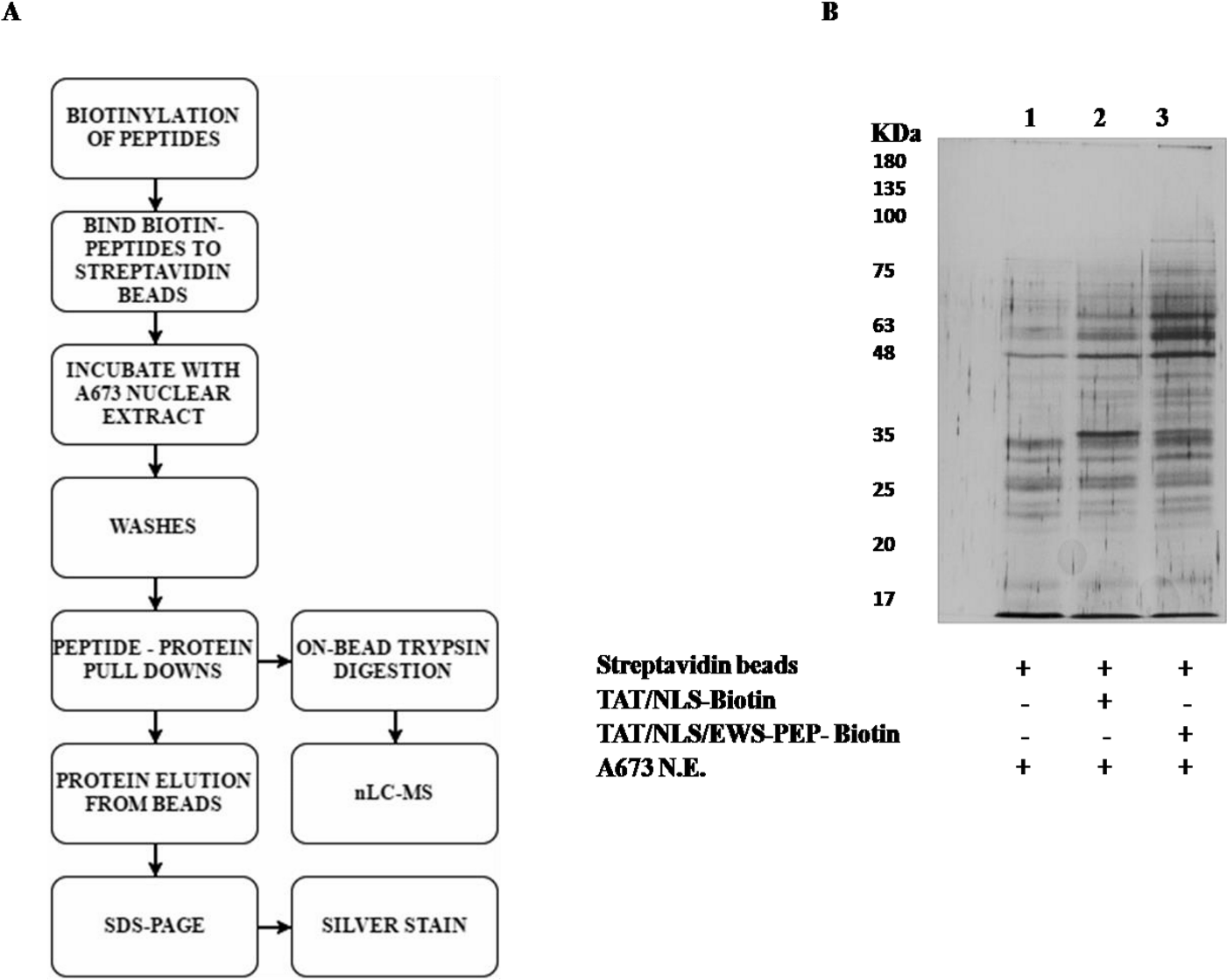


**Supplementary Figure 5**: Biotin-Peptide pull down assay using A673 nuclear extracts (N.E).

**(A)**: Workflow of peptide-protein pull down followed by nLC/MS/MS analysis

**(B)**: Representative silver stained gel depicting the Co-IP complex derived from A673 cells.

# Supplementary Table 1: List of Peptides and their amino acid sequences

| **Peptide Name** | **Number of Amino**  **Acids (a.a)** | **Peptide Sequence** |
| --- | --- | --- |
| EWS-PEP | 30 | PSQYSQQSSSYGQQNPSYDSVRRGAWGNNM |
| TAT/NLS | 19 | GRKKRRQRRRPQPKKKRKV |
| TAT/NLS/E WS-PEP | 49 | GRKKRRQRRRPQPKKKRKVPSQYSQQSSSYGQQNPSY  DSVRRGAWGNNM |

# Supplementary Table 2: Proteins identified in biotinylated peptide pull downs

| **TAT/NLS** | **COMMON PROTEINS** | **TAT/NLS/EWS-PEP** |
| --- | --- | --- |
| C1QBP_HUMAN Complement component 1 Q subcomponent-binding protein,  mitochondrial | POTEE_HUMAN POTE  ankyrin domain family member  E | TBB3_HUMAN Tubulin  beta-3 chain |
| H4_HUMAN Histone H4 | RS23_HUMAN 40S ribosomal protein S23 | TBA1B_HUMAN Tubulin  alpha-1B chain |
| RL23A_HUMAN 60S  ribosomal protein L23a | TBB5_HUMAN Tubulin beta chain | TBB1_HUMAN Tubulin  beta-1 chain |
| HNRPL_HUMAN  Heterogeneous nuclear ribonucleoprotein L |  | H2AV_HUMAN Histone H2A.V |
| RAB3C_HUMAN Ras-related protein Rab-3C |  | NPM_HUMAN  Nucleophosmin |
| RL13A_HUMAN 60S  ribosomal protein L13a |  | EF1D_HUMAN Elongation factor 1-delta |
| MYH9_HUMAN Myosin-9 |  | H31T_HUMAN Histone  H3.1t |
| RL14_HUMAN 60S ribosomal protein L14 |  | ENPL_HUMAN  Endoplasmin |
| PSD3_HUMAN PH and SEC7 domain-containing protein 3 |  | RL13_HUMAN 60S  ribosomal protein L13 |
| PESC_HUMAN Pescadillo homolog |  | ZN507_HUMAN Zinc finger protein 507 |
| EFHD1_HUMAN EF-hand  domain-containing protein D1 |  | RL17_HUMAN 60S  ribosomal protein L17 |
| TLL1_HUMAN |  | HNRPC_HUMAN  Heterogeneous nuclear ribonucleoproteins C1/C2 |

# Supplementary Table 3: List of primers

| **GENE** | **DIRECTION** | **SEQUENCE** |
| --- | --- | --- |
| CDH1 | FORWARD | AGGCCAAGCAGCAGTACATT |
|  |  |  |
|  | REVERSE | ATTCACATCCAGCACATCCA |
| CTNNA1 | FORWARD | GGGGATAAAATTGCGAAGGAGA |
|  |  |  |
|  | REVERSE | GTTGCCTCGCTTCACAGAAGA |
| CTNNB1 | FORWARD | AGCTTCCAGACACGCTATCAT |
|  | REVERSE | CGGTACAACGAGCTGTTTCTAC |
| CDH2 | FORWARD | TGGGAATCCGACGAATGG |
|  |  |  |
|  | REVERSE | TGCAGATCGGACCGGATACT |
| VIM | FORWARD | GAGAACTTTGCCGTTGAAGC |
|  |  |  |
|  | REVERSE | TCCAGCAGCTTCCTGTAGGT |
| ACTA2 | FORWARD | CGAGATCTCACTGACTACCTCATGA |
|  |  |  |
|  | REVERSE | AGAGCTACATAACACAGTTTCTCCTTGA |
| GLI1 | FORWARD | CAGGAACCTTCCTACCAGAGTCC |
|  |  |  |
|  | REVERSE | GTGCTGCTGCCTATGTGAAG |
| NKX2.2 | FORWARD | AACCCCTTCTACGACAGCAGC |
|  | REVERSE | CTCCTTGTCATTGTCCGGTGA |
| CCND1 | FORWARD | GGCGGAGGAGAACAAACAGA |
|  |  |  |
|  | REVERSE | TGAGGCGGTAGTAGGACAGGA |
| C-MYC | FORWARD | AGCAGCAGCAGAGCGAGCT |
|  |  |  |
|  | REVERSE | CACCGCCGTCGTTGTCTC |
| NR0B1 | FORWARD | CAAATGCTGGAGTCTGAACATCA |
|  |  |  |
|  | REVERSE | ACTGGAGTCCCTGAATGTACTTCAC |
| IGF1 | FORWARD | ATGGGAAAAATCAGCAGTCTTCC |
|  |  |  |
|  | REVERSE | AGATGCGAGGAGGACAT |
| EZH2 | FORWARD | CGAGAGTGTGACCCTGACCTCT |
|  |  |  |
|  | REVERSE | GCCTGCCACGTCAGATGG |
| CD99 | FORWARD | AGAGCAGAGATGGAGGCCTTCT |
|  |  |  |
|  | REVERSE | CCCAGCAACAAGCAAAGCA |
| FOXO1 | FORWARD | GATCTACGAGTGGATGGTCAAG |
|  |  |  |
|  | REVERSE | CTTGCTGTGTAGGGACAGATTA |
| IGFBP3 | FORWARD | CAGAGCACAGATACCCAGAAC |
|  |  |  |
|  | REVERSE | GGACTCAGCACATTGAGGAA |
| LOX | FORWARD | GTACTCTGACGACAACCCTTATT |
|  |  |  |
|  | REVERSE | GAAGTAGCCAGTGCCGTATC |
| GAPDH | FORWARD | GTGAAGGTCGGAGTCAACG |
|  |  |  |
|  | REVERSE | TGAGGTCAATGAAGGGGTC |

# FULL LENGTH BLOTS
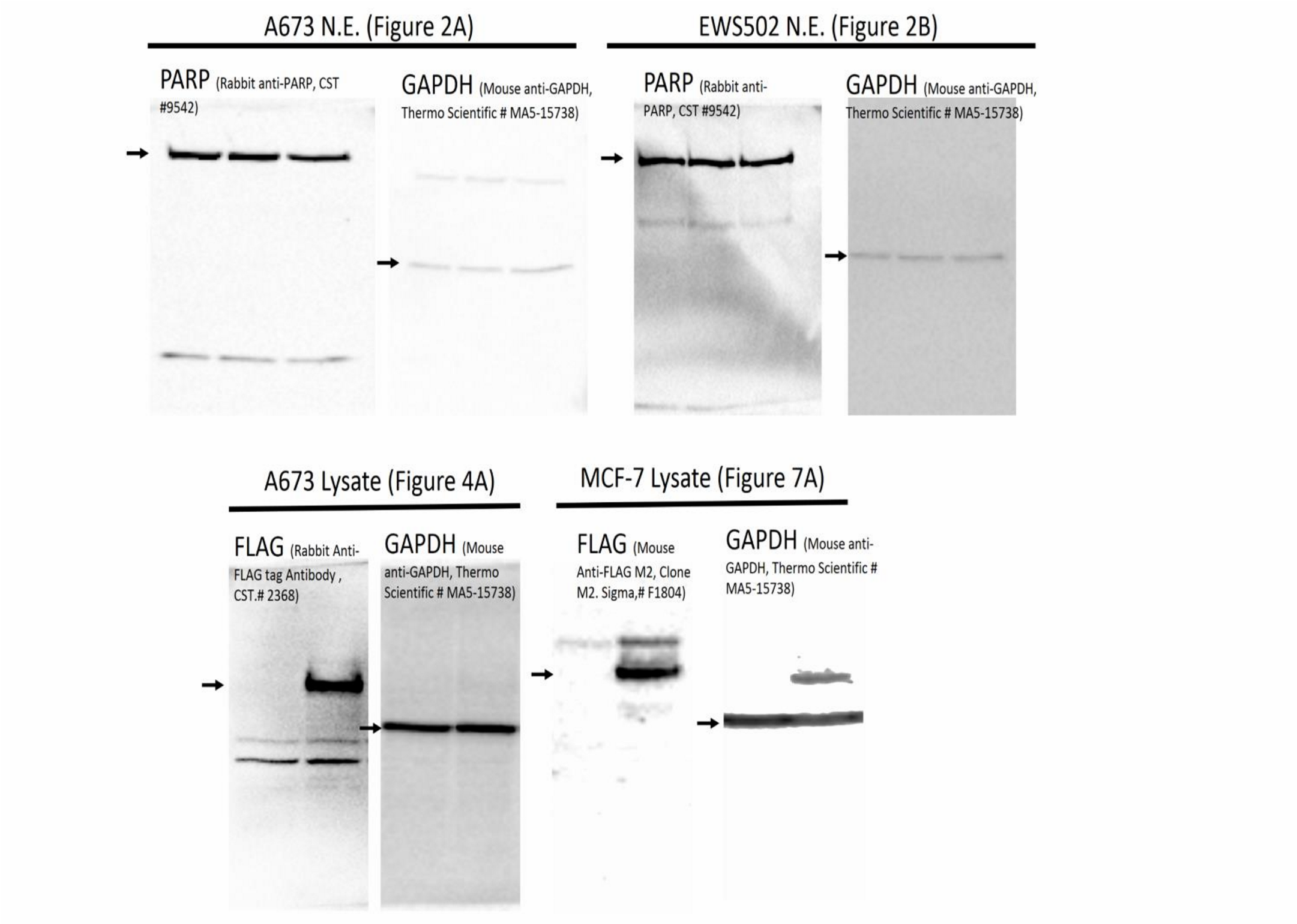

Supplement: Supplementary file 1 — Supplementary Information [file 41598_2017_7482_MOESM1_ESM.docx]
